# Queer quit: a pilot study of a smoking cessation programme tailored to gay men

**DOI:** 10.1186/1471-2458-14-126

**Published:** 2014-02-06

**Authors:** Maria Dickson-Spillmann, Robin Sullivan, Benedikt Zahno, Michael P Schaub

**Affiliations:** 1 Swiss Research Institute for Public Health and Addiction, Konradstrasse 32, 8031 Zurich, Switzerland; 2 Checkpoint Zurich, Konradstrasse 1, 8005 Zurich, Switzerland

**Keywords:** Smoking cessation, Homosexuality, Pilot project

## Abstract

**Background:**

The prevalence of cigarette smoking among adult gay males is higher than that of heterosexuals. There is a need for interventions adapted to gay culture. We conducted a pilot study using a modified version of a British smoking intervention programme tailored to gay men in Switzerland. As the main outcome, we assessed point prevalence smoking abstinence six months following programme attendance.

**Methods:**

Seventy gay smokers attended seven weekly sessions in groups (*median size* = 5) taught by gay facilitators. A quit day was set in session 3. Integral components of the intervention were: discussing nicotine replacement therapy, performing carbon monoxide tests and forming ‘quit teams’. Seven-day point prevalence smoking abstinence, mental and physical health and the frequency of alcohol and drug use were assessed at baseline, in session 7 and at a six-month follow-up.

**Results:**

Point prevalence abstinence significantly increased throughout the study (*p* = .00). At six months, 20 participants (28.6%) reported smoking abstinence over the previous 7 days. We observed increases in participants’ mental health between baseline and the six-month follow-up (*p* = .00). Participants who dropped out during the programme or were lost to follow-up smoked more cigarettes and were more nicotine dependent than the participants who were retained throughout the study duration (*p* ≤ .05).

**Conclusions:**

This smoking cessation programme for gay men produced rates of point prevalence abstinence that were similar to interventions for non-gay groups. The programme presented an opportunity for gay men to quit smoking and interact with other gay non-smokers. Our results confirm the need to test this programme more systematically with a view toward implementing it on a larger scale in Switzerland.

**Trial registration:**

Current Controlled Trials ISRCTN36851118 (02 October 2013).

## Background

Studies from Switzerland and other countries have shown that the smoking prevalence among gay men is higher than that of heterosexual men, even after controlling for demographic variables [1]. Prevalence estimates range between 14–44% for heterosexual men and 25–49% for homosexual men [2-6]. Although homosexuals have been identified as a vulnerable population regarding the health risks of smoking and the need for culture-sensitive interventions has been acknowledged [7], such interventions have rarely been studied.

One study evaluated a seven-week group smoking cessation programme adapted for gay men, provided by the UK’s Gay Men’s Health Charity (GMFA) and based on a smoking cessation programme approved by the National Health Service [8]. The pillars of this programme were group work, nicotine replacement therapy (NRT) and peer support. ‘Quit cells’ of three to four participants were created to promote partnered support. Assertiveness exercises were used to enhance participants’ resistance to temptations. The results showed that 76% (*n* = 44) of the men who had set themselves a quit date in the third week as part of the programme remained non-smokers until the end of the programme. Long-term smoking abstinence, however, was not assessed.

Swiss gay smokers expressed widespread intentions to quit smoking and a high interest in a culturally specific smoking cessation programme [9]. The smokers in this study reported that within the gay community, smoking and going out to gay venues ‘belonged’ together. The participants feared that quitting smoking, without being provided appropriate support, would mean having to abstain from the gay scene and possibly losing community membership. Thus, the participants considered it important that any successful programme help to build a non-smoking social network that would support them beyond the end of the programme. Additionally, it was found that gay healthcare organisations played an important role in communicating the risks of smoking to gay men and that such communication may draw men to cessation services.

In this article, we report the outcomes of a pilot study evaluating a smoking cessation programme for gay men. This programme is based on a similar program tested in the UK [8]. Our primary aim was to assess the success of the programme in terms of long-term point prevalence abstinence. Furthermore, we expected that by increasing participants’ skills in coping with situations of smoking temptation, participants’ ability to resist other drugs and alcohol would also be enhanced, and thus consumption of these substances would be reduced. We also intended to evaluate the beneficial effects of programme participation on depression and anxiety scores, two risk factors for decreased mental health that are widespread among homosexual men [10]. Thus, as secondary outcomes, we wanted to evaluate the programme regarding changes in mental health, physical health and other substance use. Qualitative feedback on programme obtained from our facilitators and participants completed our evaluation. From our findings, we hoped to gain information on the potential success of this programme to facilitate smoking cessation and improve other health outcomes as well as to assess its attractiveness to the target population. Given these positive evaluations, the programme might be more rigorously tested and possibly implemented on a larger scale in Switzerland.

## Methods

### Study design

As this is a pilot study, we did not include a control group. All participants underwent the same procedure. We aimed at a minimum of five groups with 15 participants each (75 participants in total).

### Programme design

In preparation for this pilot study, a personal meeting between the group work manager at the GMFA and the research manager at the Swiss Research Institute for Public Health and Addiction (ISGF) was held to share research intentions and practical experiences with the programme. Following this meeting, two programme manuals were kindly provided by the GMFA: one for facilitators and one for participants. The manuals were translated into German by a bilingual employee of the ISGF. While we maintained the programme structure, we undertook minor modifications to the educational content. These revisions included updating the information on prescription medication used to treat smoking addiction in session 1 (e.g., adding varenicline) or adapting smoking prevalence statistics to Switzerland. Table 1 summarises the content of each session.

**Table 1:** Main topics of each session (slightly modified from the GMFA manual)

**Session**	**Main topics**
Session 1	Facts about smoking and smoking cessation
	What can you expect when you quit smoking?
	Minimise the side effects – NRTs and prescription medication
	Contraindications for prescription medication
	What about prescription medication and HIV anti-retroviral therapy?
	The issues of combination usage of NRT and prescription medication
Session 2	Are you ready to stop smoking?
	Carbon monoxide and what it does to you
	Carbon monoxide monitor
	Stop Smoking action plan
	Smoking diary
	Preparing for Quit Day
Session 3	Quit Day
	Quit Team Contact Sheet
Session 4	Inexpensive holistic stop-smoking ideas
Session 5	Weight gain issues
Session 6	Your support network
Session 7	Celebration

The programme consisted of seven weekly closed-group sessions, each lasting 2.5 hours. In line with the GMFA’s programme, sessions 1 and 2 of the programme had an educational focus. These sessions were mainly managed by the facilitator. To support participants in seeking medication, a letter addressed to their general practitioners was made available announcing the participants’ attendance in the programme and requesting a prescription of NRT or other prescription medicine. Smoking cessation at session 3 (‘quit day’) was mandatory for all participants. From ‘quit day’ in session 3 onwards, priority was given to the programme’s social component. Peer support was reinforced by shifting from vertical (facilitator) to horizontal peer support (quit teams). Group discussions were initiated, and the quit teams were encouraged to engage in leisure time activities together until and beyond the end of the course.

Carbon monoxide (CO) measurements were taken in each session using a breath carbon monoxide monitor (PiCO Smokerlyzer; Bedfont Scientific Ltd., Harrietsham, Maidstone, Kent, UK) to confirm smoking cessation, monitor smoking abstinence and visualise physical health improvements. In addition, participants indicated the number of cigarettes smoked in the previous 7 days and (if any) the type and the number of units used of nicotine replacement therapy during the previous week. These assessments were included as part of the programme to make participants aware of their progress.

### Setting

The programme was delivered by four facilitators who were recruited from a gay health centre in Zurich (Checkpoint). The facilitators were between 25 and 45 years of age, male, gay, and had previous teaching experience in a psychosocial context. All facilitators were non-smokers. To promote consistency across the groups, facilitators were instructed to follow the programme manual for facilitators. Furthermore, in preparation for their task, each future facilitator co-taught the programme together with the head facilitator who had long-standing experience in teaching smoking cessation classes. The programme took place in conference rooms across the city of Zurich on weekday evenings. The programme ran nine times between January 2009 and June 2010.

### Six-month follow-up

Six calendar months following session 7, each participant was provided with an electronic follow-up form that could be filled in and returned online or printed out and sent back via postal mail. Non-responders received a reminder every two weeks following the initial mailing together with an invitation to perform the follow-up via telephone. Follow-ups, which took place between September 2009 and November 2010, were managed by a student employee of the ISGF.

### Ethical aspects and informed consent

This pilot study was performed in line with the 2004 Declaration of Helsinki. The study design was publicly available on the website of the Swiss Tobacco Control Fund. (http://www.bag.admin.ch/tabak_praevention). Following clarification with the Cantonal Ethical Committee of Zurich, the present study did require formal ethical approval as we did not administer drugs or perform the study in a hospital, nursing home or institution of justice (http://www.kek.zh.ch). Informed consent was obtained from the participants prior to their participation in the study. Participants were asked to present the signed form to the facilitators at the first session.

### Participants

Gay male smokers older than 18 years with strong intentions to quit were recruited for the study. Based on advice from the GMFA’s group work manager, we created diversified groups of participants from various backgrounds to produce more representative results and to reduce the likelihood of participants cheating or having mental blockades caused by social pressure. Advertisements were placed in local gay media and on websites; leaflets containing information about the course were left at gay bars in the city of Zurich. Moreover, an animated website was designed (http://queer-quit.ch) that included additional information about the course, the subject of smoking and the enrolment procedure. Potential participants were invited to attend an information session at Checkpoint in which information concerning the smoking prevalence in gay men, results from the programme in the UK and details about the current programme were presented. At the end of the information session, potential participants had the opportunity to sign up for the study.

### Measures and instruments

To assess the main outcome, participants were asked in session 7 and at the six-month follow-up whether they had smoked during the previous seven days. Sociodemographic information (date of birth, nationality, education), age at first cigarette, number and type of previous quit attempts and the Fagerstrom Test for Nicotine Dependence (FTND) [11] were collected in session 1. Data on mental and physical health and 30-day point prevalence of drug and alcohol use were collected in sessions 1 and 7 and at the six month follow-up using the German short version of the Beck Depression Inventory (BDI-V) [12,13], the Beck Anxiety Inventory (BAI) [14,15], the Short-Form Health Survey 12 (SF-12) [16,17] and a shortened version of the European Addiction Severity Index [18].

To evaluate the programme and determine possible barriers and enablers for smoking cessation, qualitative interviews were conducted with the facilitators, and the participants were asked to complete a short voluntary feedback form at the end of the programme.

### Data preparation and analysis

In the course of data preparation, we recoded FTND response categories to indicate that a higher value meant higher nicotine dependence, and we created a sum score for every respondent. In accordance with clinical research, where BDI and BAI scores are frequently reported in terms of sum scores, for our sample description we also calculated sum scores for these measures. For the analysis of BDI and BAI as secondary outcomes of the programme, however, we used the mean BDI and BAI values of those participants with no more than two (10%) missings. Using the two SF-12 subscales, a mental and a physical component score were created according to instructions [19]. Alcohol and drug usage was coded into different categories based on frequency of use that ranged from “never” to “3 or more times a day”.

This study is based on an intention-to-treat design. For the evaluation of our main outcome of point prevalence smoking abstinence, those participants who did not provide follow-up data were counted as relapsed smokers. To investigate whether point prevalence abstinence increased over time, we applied generalised estimating equation (GEE) analyses to account for correlations between repeated observations. The dichotomous variable “7-day smoking abstinence” was entered into the model as the dependent variable, with “time” (i.e. baseline, session 7 or six-month follow-up) as the predictor. GEE analyses were also applied to secondary outcomes.

We analysed baseline differences between dropouts at the six-month follow-up and those who stayed in the programme using t-tests and X^2^-tests. All analyses were conducted in STATA 12 SE (College Station, Texas, USA).

Open-ended, qualitative feedback regarding the programme was coded into different categories by one rater; this categorisation was then reviewed by another rater. In cases of disagreement, the raters discussed both alternatives until they reached a consensus.

## Results

### Participant flow

Seventy participants were recruited for the study. At the first session, the median group size was five (*Min* = 2, *Max* = 21). Session 3 was attended by 59 participants, which represented 84.3% of all participants who had attended the first session. Forty-seven men attended the last session (67.1%). Thirty-eight participants were reached at the six-month follow-up (54.3%). Complete datasets including baseline, session 7 and the six-month follow-up were available for 35 participants (50.0%).

### Participant characteristics

Table 2 summarises the baseline demographics, health characteristics and smoking habits of the participants. As defined by the FTND which ranges from 0 to 10, the study sample consisted of medium-dependent smokers (*M* = 4.1, *SD* = 2.3), who had undertaken more than three quit attempts before beginning the programme. Our sample was older than a Swiss sample of 477 gay men in a health survey (35 years) [6] and did not contain as many university-educated individuals (38.6% vs. 12.9%), but rather contained more participants with vocational training (28.4% vs. 51.5%). Depression scores according to the BDI-V were higher in our sample (*M* = 28.75, *SD* = 15.19) than in a representative sample of the German male population (*M* = 18.8, *SD* = 13.5) [12].

**Table 2:** **Sample description (*N*** **= 70)**

**Variable**	**n (%)**
Age *(M, SD)*	42.96 (9.66)
Nationality	
Switzerland	51 (72.9)
Germany	9 (12.9)
Other	10 (14.3)
Education	
Compulsory education	0 (0.0)
Vocational education (apprenticeship)	36 (51.5)
General education (Matura certificate)	4 (5.7)
University of applied science	20 (28.6)
University	9 (12.9)
Other	1 (1.3)
Smoking cessation	
Quit attempts *(M, SD)*	3.38 (2.49)
NRT* experienced	36 (51.4)
Prescription medication** experienced	13 (18.6)
Smoking habits	
Fagerstrom Nicotine Tolerance score *(M, SD)*	4.13 (2.33)
Age at smoking initiation *(M, SD)*	18.58 (4.14)
Years of smoking *(M, SD)*	22.83 (9.30)
Drug use	
Alcohol: 3–4 times per week	19 (27.1)
Cannabis: at least once in last 30 days	32 (45.7)
Cocaine: at least once in last 30 days	8 (11.4)
Party drugs: at least once in last 30 days	17 (24.3)
Health	
HIV positive	9 (12.9)
Mental component scale *(M, SD)*	55.78 (8.11)
Physical component scale *(M, SD)*	52.48 (6.06)
Depression	
BDI-V *(M, SD)*	28.75 (15.19)
Lifetime diagnosis	13 (18.6)
Anxiety	
BAI *(M, SD)*	9.31 (6.83)
Lifetime diagnosis	6 (8.6)

*Nicotine replacement therapy.

**Prescription medication used to treat smoking addiction (e.g., bupropion).

### Self-reported point prevalence abstinence, CO measurements and use of NRT

Of the participants who attended session 7, one reported having smoked a cigarette during the previous week. All other participants reported having been smoke-free (65.7% abstinent participants on an intention-to-treat basis). The mean CO score at session 7 was 2.72 (*SD* = 1.56). At the six-month follow-up, 20 participants reported that they had not smoked in the previous seven days (28.6% of the original 70 participants). According to the GEE analyses, “time” had a significant impact on abstinence rates (*OR* 2.01, *CI* 1.60 - 2.52, *p* = .00).

In session 3, 28 of 59 (47.5%) participants reported using NRT. Varenicline tablets were the predominant therapy (used by 11 participants), followed by 2 mg gum (5), 4 mg gum (3) and 16 h patches (3). In session 7, 18 of 47 (38.3%) participants used NRT, with tablets still being the most frequently used therapy (9 participants), followed by the 2 mg gum (4).

### Changes in substance use and mental and physical health

The GEE analyses revealed changes over time in depression (*b* = -.16, *SE* = .04, *p* = .00, *CI* [-.24] – [-.08]), anxiety (*b* = -.07, *SE* = .02, *p* = .00, *CI* [-.12] – [-.03]) and the mental component score of the SF-12 (*b* = 2.09, *SE* = .67, *p* = .00, *CI* 0.78 – 3.41). Depression and anxiety scores decreased by 0.29 and 0.16 points, respectively, while the mental component score increased by 1.39 points. In contrast, “time” did not affect frequency of alcohol and drug use in the previous 30 days, nor did it affect physical wellbeing (all *p* > .05). No differences in depression and anxiety scores were found between participants who were abstinent at six months and those who had relapsed (*p* > .05).

### Differences between retained participants and those lost to follow-up

Participants who were available for the six-month follow-up and those who were not differed with regard to the number of daily cigarettes smoked at baseline during the previous month. Retained participants smoked 2.11 (*SD* = 0.76) cigarettes, while those lost to follow-up smoked 2.56 (*SD* = 0.89) (*p* = .02). Similarly, the latter participants were slightly more nicotine dependent at baseline (*M* = 5.24, *SD* = 2.50) than those who were available at follow-up (*M* = 4.11, *SD* = 2.07, *p* = .05). Similar differences in these two smoking-related variables were observed between the participants who attended session 7 and those who did not (results not shown). No differences were found between the retained participants and drop-outs with regards to other smoking-related variables, sociodemographic and health characteristics or substance use at either session 7 or follow-up.

### Participants’ feedback and interviews with facilitators

At the end of the programme, 36 participants (77 of those present at session 7) completed the short feedback form. Table 3 shows the results of the closed-end questions in the participant satisfaction survey. When asked through open-ended questions for positive experiences in the programme, 13 participants mentioned the general points of ‘group dynamics’ and ‘group experience’. More specific points included ‘group communication’, which was mentioned by 13 participants, ‘control’ (2 participants) and ‘having a common aim’ (2 participants). Four participants reported that they perceived the regularity of meetings as positive. In relation to specific programme elements, two participants found the CO test and two participants found the information materials to be particularly helpful. Only one person explicitly mentioned the fact that the programme was only for gay participants as being particularly positive. When asked for negative aspects of the programme, seven participants noted that they had problems with the skills trainings, and two mentioned that they had hoped for more group discussions.

**Table 3:** **Program evaluation by the participants (*N*** **= 36)**

**Item**	**Very much agree**		**Partly agree**		**Don’t agree**	
	**n**	**%**	**n**	**%**	**n**	**%**
I am happy with the program content.	26	72.2	10	27.8	0	0.0
The program was easy to understand.	34	94.4	2	5.6	0	0.0
I can use the program content in my everyday life.	31	86.1	2	13.9	0	0.0
The program structure helped me achieve smoking cessation.	29	80.6	6	16.7	1	2.8

The interviews with the facilitators suggested that they would have preferred the use of NRT and prescription medication to be explained by a general practitioner with more knowledge about side effects and contraindications. The facilitators considered group sizes of 10 to 12 participants to be optimal, particularly for group discussions.

## Discussion

We evaluated a smoking cessation programme tailored to gay men, a group with a high smoking prevalence. At the end of the programme, two-thirds of participants reported smoking abstinence. Self-reported abstinence by the participants was validated by the presence of low CO levels (< 3 ppm) observed at the last session [20,21]. At the six-month follow-up, more than one-quarter of participants reported smoking abstinence during the previous week. At the same time, we observed improvements in the mental health of all participants during the study period, both in those who quit smoking and those who relapsed. Participation in the programme did not lead to lower consumption of illegal drugs and alcohol. The programme was well accepted by both the facilitators and the participants. Use of NRT decreased by nearly 10% from session 1 to session 7. Qualitative responses showed that the participants particularly appreciated the group dynamics and the group experience of the course. This finding underlines the importance for our participants to experience smoking cessation together with other gay men, which is a desire expressed in the context of a previous study [9].

In the UK study [8], 58% of all participants who had attended the first session were abstinent at the last session. Our abstinence rate at session 7 of 66% is therefore comparable to that of the UK study. Our result of abstinence in 28.6% of participants at six-month follow-up is similar to that of other group intervention studies of smoking cessation in non-gay samples: Huber and Gastner [22] reported 23 and 29% abstinence in participants, without and with NRT, respectively; Schmitz et al. [23] observed 22%, and 30% abstinence without and with bupropion, respectively; and Romand, Gourgou and Sancho-Garnier [24] reported 20% abstinence without medication. Considering that our sample was more burdened with depression than the general male population and that negative affect could be a barrier to smoking cessation [25,26], the similarity of our abstinence rate with the rates observed in other studies is remarkable.

The observed improvement in mental health could be attributed to the fact that, through attending the programme, all participants had the opportunity to actively address their nicotine addiction and to acquire the knowledge necessary to control this addiction. This process was perhaps more important for participants’ mental health than their actual success in quitting. Contrary to our expectation, the programme did not affect consumption of other drugs. This might be because the programme (e.g. the assertiveness exercises) was specific to smoking tobacco, because the participants may not have considered their use of other drugs as being problematic, or may have perceived the consumption of other drugs as a separate issue.

Our study has some limitations that merit discussion. Due to the absence of a control condition in our pilot study and our rather small sample, we are not able to draw conclusions regarding the efficacy of the programme for the general population. Participants who dropped out (*i.e.* no attendance at session 7 or lost to follow-up) at baseline smoked more cigarettes per day and were more nicotine-dependent than those who completed the study. It may be that heavier smokers generally encounter more difficulties when attempting smoking cessation [27]. However, this outcome could also indicate that the programme – through its structure or its content – addressed the needs of lighter rather than heavier smokers. Because of the absence of a control condition, we cannot assess the influence of pre-existing intentions to quit smoking on our participants, which were possibly stronger than those of their peers who did not attend the programme and, as such, could have enhanced participants’ success regardless of program participation.

We are unable to distinguish between the effects of the general programme structure, those that occurred through the specific adaptations made for the gay population and those that occurred through the implementation (e.g. gay facilitators, gay-only participants) on smoking behaviour. We do not know whether our study participants would have benefited from the programme to the same extent if heterosexual participants had also participated, if heterosexual facilitators had taught the program or if the content was not specific to the gay culture. A previous study found that cessation rates were nearly identical between homosexual (59%) and heterosexual (57%) male participants [28] at the last session of a non-tailored smoking cessation programme; rates comparable to those we observed. Follow-up results were not reported for that study. Thus, the effect of tailoring the program specifically to the homosexual population remains unknown. Qualitative remarks, however, indicated that many participants appreciated the atmosphere of open discussion at the sessions. It is likely that such an atmosphere more easily evolved through the match between facilitators’ and participants’ sexual orientation.

Further research is necessary to identify the specific programme components that contributed in a statistically significant way to the effectiveness of this pilot intervention. Prior research has shown that general problem-solving elements (skills training, relapse prevention and stress management) are likely to be beneficial, as is intra-treatment social support [29]. The next step will be to establish the efficacy of the programme in the context of a randomized-controlled trial. If efficacy can be proven, the programme will be implemented nationwide.

Future research could focus on evaluating the programme in homosexuals with HIV. Due to the double physical burden, smokers with HIV are at enhanced risk for pulmonary, cardiovascular and bone diseases, as well as malignancies and generally lowered immune responses [30]. In addition, smoking is associated with lower adherence to highly active antiretroviral therapy [31]. Thus, promoting smoking cessation in HIV-infected smokers is a particular concern.

## Conclusions

More than a quarter of our participants remained smoking-abstinent for at least six months. The participants and the facilitators provided positive evaluations of the programme that particularly emphasised the group experience. Thus, for homosexual men who can afford the time to commit to seven weekly sessions, this programme is a promising way to quit smoking and to develop relationships with other non-smoking homosexuals. A randomized-controlled trial of the programme and its implementation at a larger scale in Switzerland will yield more generalizable results on its success. A particular focus of research related to the programme will be smoking cessation in homosexuals with HIV.

## Competing interest

The authors declare that they have no competing interests.

## Authors’ contributions

MDS performed statistical data analysis and drafted the manuscript. RS translated the manuals, coordinated the study and performed follow-up interviews. BZ supervised the implementation of the programme at Checkpoint and revised the programme manuals. MS contributed to the study design and to the final draft of the manuscript. All authors read and approved the final manuscript.

## Pre-publication history

The pre-publication history for this paper can be accessed here:

http://www.biomedcentral.com/1471-2458/14/126/prepub
